# Optimal extent of lymph node dissection in gastric cancer

**DOI:** 10.3389/fsurg.2022.1093324

**Published:** 2022-12-29

**Authors:** Zsolt Varga, Péter Kolozsi, Kitti Nagy, Dezső Tóth

**Affiliations:** Department of Surgery, University of Debrecen, Debrecen, Hungary

**Keywords:** gastric cancer, lymph node, lymphadenectomy, D1D2, gastrectomy, laparoscopy

## Abstract

Gastric cancer still remains a major cause of cancer-related deaths globally. Stage-adapted, individualized treatment is crucial to achieving optimal oncological outcomes. Postoperative morbidity and accurate nodal staging are heavily influenced by the extent of lymph node dissection. On one hand, insufficient lymphadenectomy may result in understaging and undertreatment of a patient, on the other hand, unnecessary lymph node dissection may result in a higher rate of postoperative complications. Approximately one-third of patients with gastric cancer undergoes an avoidable lymph node dissection. Many of the recent treatment updates in the management of gastric cancer have a major influence on both surgical and oncological approaches. Currently, a wide range of endoscopic, minimally invasive, and hybrid surgical techniques are available. The concept of sentinel node biopsy and utilization of the Maruyama Computer Program are significant components of stage-adapted gastric cancer surgery. Likewise, centralization and application of national guidelines, widespread use of neoadjuvant therapy, and the stage migration phenomenon are serious concerns to be discussed. Our goal is to review the available surgical strategies for gastric cancer, with a primary focus on lymphadenectomy.

## Introduction

According to the recent GLOBOCAN 2020 estimation (1), gastric cancer is the fifth most common cancer worldwide. The number of new cases was estimated to be 1 089 103 with 768 793 deaths. The diagnosis of gastric cancer is frequently made at an advanced stage, resulting in a high mortality rate. Countries with the highest incidence and mortality are located in East Asia, Eastern Europe, and South America. The incidence rates in males are more than two-fold higher (15.8 and 7.0 per 100 000) than in females (1). Economic development has contributed to the global reduction in the prevalence of H. pylori, a major factor for gastric cancer, as well as eradication therapy. Additionally, gastroduodenoscopy screening programs in Asia have led to a significant decline in the mortality of this disease (2). There is a well-known positive association between gastroesophageal reflux disease (GERD) and proximal gastric cancer (3). Despite the current promising tendency, the dietary habits and aging of the population in developed countries might reverse these trends. Moreover, in Western societies, there has been a gradual decrease in the incidence of the distal, intestinal type of gastric cancer, and an increase in the proximal, diffuse type (4). In 2014 the Cancer Genome Atlas Research Network identified and published four molecular subtypes of gastric cancer: Epstein-Barr virus positive, microsatellite unstable tumors, genomically stable, and chromosomally unstable tumors (5). In recent years, novel diagnostic tools utilizing algorithmic analysis in digital imaging (6), as well as liquid biopsy techniques, have evolved.

It has been more than 140 years since Theodor Billroth's (1829–1894) first successful gastric resection for cancer in 1881. Regardless of the scientific and technological advancement, the development of a multimodal treatment approach using resection (surgical or endoscopic) is still the foundation of curative management in gastric cancer (7). Stage-adapted, individualized treatment is crucial to achieving optimal oncological outcomes. The latest, 8th edition of the TNM Classification of Malignant Tumours (8) is most frequently used to stage patients. Diagnostic modalities including contrast-enhanced chest-abdomen-pelvis CT, esophagogastroduodenoscopy, endoscopic ultrasound, and explorative laparoscopy are all helpful in the staging process. The latter procedure, along with peritoneal lavage is recommended for stage IB–III patients before surgical resection (9). The clinical stage will determine the treatment approach, which is decided by a competent multidisciplinary tumor board. There is however a concerning amount of variation among treatment guidelines, depending on the region (7). Generally, clinically staged T1N + M0 and T2–T4aN(any)M0 gastric cancer requires surgical resection with adequate lymphadenectomy, together with perioperative or adjuvant chemotherapy. Surgery aims to achieve local control through free surgical resection margins and clearance of regional lymph nodes.

In 1973 the Japanese Research Society for Gastric Cancer established the blueprint that standardized lymph node dissection in gastric cancer (10). In this manual, they recognized 16 distinct lymph node stations based on their anatomical location, and created a system to measure the extent of lymphadenectomy, namely D1, D2 and D3. Since then, the guideline has been revised multiple times. The latest, 5th edition was published in 2018 (11) where D-levels are now defined by the location of the tumor and the surgery performed. As a simplification, D1 lymphadenectomy implicates the removal of the perigastric nodes plus those along the left gastric artery (station 1–7), while D2 implies the removal of D1 nodes, plus nodes along the common hepatic and splenic artery, and the coeliac trunk. D1 + lymphadenectomy is defined according to the type of gastrectomy. D3 lymphadenectomy includes dissection of all D2 lymph node stations, extended by well-defined abdominal paraaortic and hepatoduodenal lymph nodes.

Postoperative morbidity and accurate nodal staging are heavily influenced by the extent of lymph node dissection. Insufficient lymphadenectomy may result in understaging and undertreatment of a patient, however, unnecessary lymph node dissection may have higher rates of postoperative complications. The optimal extent of lymph node dissection has been debated over the last decades. The Eastern rationale focuses on more accurate staging and better locoregional control, whereas early Western data showed notable morbidity and mortality by this procedure. This review aims to summarize the current guidelines and evidence on this subject.

### Lymph node metastases

Lymph node (LN) involvement is one of the most important prognostic factors for gastric cancer. Conventional preoperative imaging techniques provide an accurate T and M stage, but there is significant uncertainty regarding the N stage. The sensitivity, specificity, and accuracy of CT scans in the detection of LN involvement are 73.1%, 50.0%, and 84.2%, respectively. Endoscopic ultrasonography performance is relatively similar with an accuracy of 68.6% and sensitivity and specificity of 66.7% and 73.7% (12).

It has been previously reported that in early gastric cancer the rate of lymph node metastasis is 2%–20% (13). Consequently, lymphadenectomy for node-negative patients bears unnecessary risks for complications. The term „early gastric cancer” (EGC) was first described by the Japanese Society of Gastroenterology and Endoscopy in 1971 (14). They then defined it as being „limited to the gastric mucosa and/or submucosa”, regardless of the lymph node status. These tumors should have a favorable prognosis, but lymph node positive patients are known to have much worse outcomes: the 99% 5-year overall survival (OS) rate for node-negative patients decreases to 73.2% in node-positive ones (15). The tumor size, depth of invasion, grade of differentiation, presence of ulceration and presence of lymphovascular invasion are known risk factors for lymph node metastases in gastric cancer (16). It is difficult to determine which patient could be spared from an unnecessarily extended lymphadenectomy, since gastric cancers can have multidirectional and complicated lymphatic flow.

### Sentinel lymph node biopsy (SLNB)

The concept of sentinel lymph node (SLN) mapping has been suggested and later implemented to identify these patients during a surgical resection (17).

The SLN is defined as the first node to receive lymphatic flow from a tumor, theoretically representing the status of the other regional lymph nodes. Their use was first described in parotid tumors and mentioned later in penile cancer, melanoma, testicular cancer, and breast cancer (18). In gastric cancer surgery, various tracers have been used: blue dye, indocyanine green (ICG), radiocolloids, and their combinations (19).

Sentinel node navigation surgery (SNNS) is a type of surgical technique that is performed according to the status of the sentinel lymph node. If the sentinel lymph node is free of metastases, gastrectomy and D2 lymph node dissection may not be necessary. The promise of this approach is the lesser extent of resection and lymph node dissection, resulting in organ preservation, faster postoperative recovery, and better quality of life (QoL) without compromising oncological safety. But this concept has yet to be proven in a clinical setting.

The application of different agents is influenced by their technical demand, visibility, cost-effectiveness, and safety. A recent systematic review and meta-analysis has shown similar pooled sensitivity rates: 82% (95%CI: 77%–86%) for blue dye, 87% (95%CI: 81%–92%) for radiocolloid tracer, 90% (95%CI: 82%–95%) for ICG, 89% (95%CI: 84%–93%) for a combination of radiocolloid with blue dye, and 88% (95%CI: 79%–94%) for a combination of radiocolloid with ICG (20). Blue dye is the most convenient and cost-effective, but its use might be limited in obese patients. The use of radioactive substances is associated with biohazard production, high costs, and high demand for specific logistical arrangements. The use of ICG seemed promising, however, suitable applications of near-infrared or fluorescence imaging have yet to be determined. Factors requiring measurement include ICG concentration, used volume, injection site, timing after injection and patient selection.

Another obstacle for intraoperative SLNB is the reliability of the pathological assessment. The Japanese JCOG0302 study was terminated due to the high (46.4%) false negative rate. The main reason for this unreliability was the single-plane frozen section. The use of interval sections, immunohistochemistry, reverse transcription polymerase chain reaction and one-step nucleic acid amplification assay have all been described (21). In the study protocol of the Korean SENORITA trial, nodes that were thicker than 4 mm were sliced at 2-mm intervals parallel to the long axis, so as not to miss macrometastasis. This promising clinical trial assessed the feasibility of laparoscopic stomach-preserving surgery with sentinel basin dissection in early gastric cancer.

The concept of sentinel basin dissection was first introduced by Miwa et al. in 2003 (22). They divided the gastric lymphatic compartments into five regions. It improved the accuracy of the conventional pick-up biopsy to 98%, however, the histological evaluation of this larger number of lymph nodes takes more time. The frequency of skip metastases in a patient with early gastric cancer was 2,8% by Lee SE et al. (23).

### Tumor control

Primary tumor control during SNNS is the key to a successful procedure. Several endoscopic and hybrid resection techniques have been published. Endoscopic submucosal dissection (ESD) has proven to be superior to endoscopic mucosal resection. The guideline of the European Society of Gastrointestinal Endoscopy (ESGE) was updated in 2022 and still recommends ESD as the treatment of choice for most gastric superficial neoplastic lesions to provide an en-bloc resection (24). Along with ESGE, the Japanese Gastric Cancer Association (JGCA) (11), European Society for Medical Oncology (ESMO) (9) and National Comprehensive Cancer Network (NCCN) (25) placed strict criteria for endoscopic resection. The NCCN and ESMO guidelines recommend endoscopic resection only in well-differentiated (G1-G2), ≤2 cm, non-ulcerated T1a lesions. There are several other cases when the JGCA guideline recommends endoscopic resection based on absolute, expanded, and relative indications. It also mentions the categories of endoscopic curability, which will determine whether the patient needs observation, additional ESD, or surgery.

There are numerous hybrid techniques published, mostly taken from the management of gastric subepithelial lesions. In 2012, Nunobe et al. published the application of laparoscopy endoscopy cooperative surgery (LECS) for lateral-spreading mucosal gastric cancer (26). Other advanced endoscopic techniques are laparoscopic-assisted endoscopic resection, endoscopically assisted wedge resection, endoscopic assisted transgastric and intragastric surgery, laparoscopic-assisted endoscopic full-thickness resection (LAEFR), the combination of laparoscopic and endoscopic approaches to neoplasia with a non-exposure technique (CLEAN-NET), and non-exposed endoscopic wall-inversion surgery (NEWS). There is profoundly limited clinical experience with these methods (27).

T1 tumors that do not meet the criteria for endoscopic resection, will require surgery, although less extensive than other gastric cancers (9). Complication rates are lower in pylorus-preserving gastrectomy, laparoscopic wedge resection, and proximal gastrectomy as compared to conventional distal or total gastrectomy. However, they can result in procedure-specific complications, eg. high rates of reflux esophagitis and anastomotic stenosis after conventional proximal gastrectomy (28). The use of jejunal interposition and double-tract reconstruction can improve nutritional parameters and anemia (29), but can be technically challenging. The short-term outcomes of the KLASS-05 trial (which randomized patients between proximal gastrectomy with double-track reconstruction and total gastrectomy with Roux-en-Y reconstruction) were comparable in the two groups (30). Another limitation of their spread is the relatively low number of patients diagnosed with early gastric cancer out of Asia. The ESMO guideline does not even mention these techniques as feasible alternatives.

In resectable, clinically staged T1N + M0 and T2–T4aN(any)M0 gastric cancer, gastrectomy with adequate lymphadenectomy is indicated to achieve local control. The JGCA recommends a resection margin of at least 2 cm for T1 tumors, and at least a 3 cm proximal margin in T2 or deeper tumors with Borrmann type I and II tumors. For Borrmann types III and IV it recommends a 5 cm proximal margin (11). The NCCN and ESMO suggest a distal gastrectomy (DG) for distal gastric cancers if safe margins can be achieved, otherwise, a total gastrectomy should be performed (TG) (9, 25). The ESMO recommends a proximal margin of 5 cm for stage IB–III gastric cancer and 8 cm for diffuse cancer when performing DG (9). When these rules cannot be satisfied, it is advisable to examine the entire thickness of the proximal resection margin by frozen section. While it seems an independent issue, the level of nodal dissection is strongly influenced by the extent of gastrectomy, and it has been extensively debated.

As for radiotherapy, there are no randomized trials were assessing the benefit of preoperative chemoradiotherapy (CRT) for non-cardia gastric cancers. The Dutch CRITICS (ChemoRadiotherapy after Induction chemoTherapy In Cancer of the Stomach) trial addressed the role of postoperative CRT (31). Patients involved with potentially resectable gastric cancer, who received induction chemotherapy followed by surgery then were randomized to postoperative chemotherapy (CT) vs. chemoradiotherapy (CRT). Postoperative compliance was poor: of the 788 patients, 478 started post-operative treatment according to protocol, 233 (59%) patients in the CT group, and 245 (62%) patients in the CRT group. Although the initial median survival after a median follow-up of 61.4 months was not significantly different between postoperative CT and CRT (43 months in the CT group and 37 months in the CRT, *p* = 0.90), per protocol analysis (32) of patients who started the allocated post-operative treatment in the trial showed that the CT group had a significantly better 5-year overall survival than the CRT group (57.9% in the CT group vs. 45.5% in the CRT group, *p* = 0.0004).

The CRITICS II trial (33) is about to evaluate the three preoperative strategies: neoadjuvant chemotherapy followed by surgery vs. neoadjuvant chemotherapy and subsequent chemoradiotherapy followed by surgery vs. neoadjuvant chemoradiotherapy followed by surgery in resectable gastric cancer.

### D1 vs. D2 lymphadenectomy

Three early European, phase III studies conducted by the British or Medical Research Council (MRC) (34), the Dutch (35), and the Italian (36) randomized control trials found that there was no early survival benefit in D2 dissection compared to D1. Interestingly, the 15-year follow-up results of the Dutch D1D2 trial showed lower locoregional recurrence and gastric-cancer-related death rates in the D2 group (37). It was preceded by the subgroup analysis of the Italian study. Degiuli et al. found that in patients with T2–T4 node-positive gastric cancer the 5-year disease-specific survival (DSS) after D2 lymph node dissection was greater than that in the D1 group (59% vs. 38%, *p* = 0.055) (36). Similarly, after a 15-year follow-up of the Italian study, disease-specific survival of patients with advanced disease and lymph node metastases was improved by the D2 procedure (38). DSS was significantly higher after D2 in pT > 1N + patients (29.4% vs. 51.4%, *p* = 0.035).

The British and Dutch studies were rightly the subjects of major criticism. The lack of survival benefit after D2 dissection is explained by the extremely high postoperative mortality in this group (13% in the British and 10% in the Dutch trial for D2 patients). In contrast, the mortality rate in the JCOG9501 study was 0.8% for D2 patients. It was likely the result of inexperienced surgeons, low-volume centers, and high rates of splenectomies and pancreatic resections in these classic trials. The 15-year follow-up Dutch data resolved this problem, showing that D2 patients without pancreatosplenectomy had a significantly higher OS than those who had D1 surgery: 35% (95% CI: 29%–42%) vs. 22% (95% CI: 17%–26%) (37). Besides, the Dutch trial enrolled 40% of patients, who had early gastric cancer, a surprisingly high proportion. In America, the famous Intergroup Trial 0116 showed an alarming snapshot: 54.3% of patients received less than D1 lymphadenectomy, and only 9.8% received a D2 procedure (39).

Meanwhile in Asia, the role of more extensive lymphadenectomies was examined. The JCOG9501 randomized controlled trial compared Japanese standard D2 and D3 (D2 + para-aortic) dissections in T2b, T3, or T4 stage gastric cancer patients. It failed to demonstrate the superiority of the extended, D3 lymphadenectomy since the 5-year OS was similar (70.3% for D3 and 69.2% for D2). The rate of morbidity was higher in the D3 group (28.1% vs. 20.9%), and mortality was very low (0.8% in both groups) (40).

The goal of lymph node dissection is also to provide adequate staging and prevent the so-called stage migration (or Will-Rogers) phenomenon. Based on the UICC and NCCN guidelines, harvesting and examining a minimum of 15 lymph nodes is required (25).

There is growing international consensus supporting the performance of gastrectomies with D2 lymphadenectomy on non-early gastric patients, especially in high-volume centers, by experienced surgeons (9).

The emerging role of perioperative chemotherapy in patients with locally advanced gastric cancer in the Western hemisphere should be noted. There is a strong recommendation for the use of neoadjuvant therapy for a patient with resectable gastric cancer stage 1B or greater (9). The effect on the lymphatic drainage of the tumors and the usefulness of all these previous findings remains unknown.

In 2006, the results of the multicentric Medical Research Council Adjuvant Gastric Infusional Chemotherapy (MAGIC) trial were published and became a landmark in perioperative systemic treatment (41). The study involved 503 patients with gastric and distal esophageal adenocarcinoma, including esophagogastric junction tumors. The recruitment lasted for 8 years. The patients on the control arm received surgery alone (*n* = 253), while patients on the experimental arm (*n* = 250) received surgery and 3 cycles of ECF (intravenous epirubicin, cisplatin, and fluorouracil) both in pre-and post-operative settings. Eventually, 104 of 250 patients (41.6%) assigned to perioperative chemotherapy completed all six cycles. The type of resection was left at the discretion of the participating surgeon, and likewise the extent of lymph node dissection. The study showed a significant improvement in oncological outcomes. The 5-year overall survival was 36.3% in the experimental group and 23% in the control group (*p* = 0.009).

The conclusions were heavily debated (42) of the long recruitment period, the inclusion of esophageal cancers, poor quality of surgery, and insufficient lymphadenectomy. Besides the low completion rate of the postoperative treatment, neither the clinical nor the pathological response to chemotherapy was not evaluated. One might presume that there is a bias towards chemotherapy, as it did no more than compensate to a certain extent for insufficient lymphadenectomy and inadequate surgery.

Another cornerstone study for perioperative oncological treatment in the West was published in 2019 (43). The FLOT4 randomized phase II/III trial has reported that the combination of docetaxel-based triplet FLOT (fluorouracil plus leucovorin, oxaliplatin, and docetaxel) was superior to standard ECF or ECX (capecitabine instead of 5-FU) regimens. The study population consisted of 716 patients with locally advanced resectable gastric (44%) or gastro-esophageal junctional (Siewert I-II-III, 56%) non-metastatic adenocarcinoma. After randomization 360 patients were assigned to the standard regimen and 356 to FLOT. Surgery was performed 4 weeks after the completion of preoperative chemotherapy. For gastric cancer, total or subtotal distal gastrectomy with D2 lymphadenectomy was performed. The 5-year overall survival was 45% in the FLOT group and 36% in ECF/ECX. It was shown that more pathologically node-negative patients were found in the FLOT group (49% vs. 41%, *p* = 0.025) and more patients had negative surgical margins in the FLOT group (85% vs. 78%, *p* = 0.0162). The superiority of FLOT therapy made ECF/ECX regimens fall out of favor for patients with excellent performance status.

D1 + lymphadenectomy is thoroughly discussed in Eastern guidelines. In the JGCA Guideline (11) that refers to a D1 lymphadenectomy plus stages 8a, 9, and 11p in total and proximal gastrectomy; D1 + No. 8a, 9 in distal gastrectomy and pylorus-preserving gastrectomy. It is noted, that for tumors invading the esophagus, No. 110 (lower thoracic para-esophageal nodes) should additionally be dissected in D1 + lymphadenectomy.

Both JGCA and ESMO Guideline recommend D1 + lymphadenectomy for cT1N0 tumors, which do not meet the criteria for endoscopic resection (hence these criteria are different in these two guidelines) (9, 11). NCCN guideline does not mention it as an option (25).

### Splenectomy and splenic hilar lymph nodes

Approximately 7.3% to 18.3% of proximal gastric cancer metastasize to the lymph nodes in the splenic hilum (44). No studies have demonstrated the advantage of prophylactic splenectomy so far. In addition, the JCOG0110 trial showed higher morbidity for the splenectomy group (30.3% vs. 16.7%) without improving survival (5-year OS rates were 75.1% vs. 76%) (45). In this study they recruited patients with T2-4N0-2M0 proximal gastric adenocarcinoma that did not invade the greater curvature.

The current JGCA guideline recommends splenic hilar lymph node (station No. 10) dissection with or without splenectomy for proximal gastric cancer invading the greater curvature (11). It suggests total gastrectomy with splenectomy for tumors located along the greater curvature and harbor metastasis to No. 4sb lymph nodes. The NCCN did not recommend routine splenectomy without direct splenic invasion or hilar lymphadenopathy (25). The ESMO guideline has no recommendations for splenectomy (9).

With the ongoing JCOG1809, the Japan Clinical Oncology Group has initiated a study to evaluate the safety of surgery involving laparoscopic and robotic dissection of the splenic hilar nodes without splenectomy.

### Maruyama computer program

The Maruyama Computer Program (MCP) was developed by Keiichi Maruyama and published in 1989 (46). It uses a database of 4,302 primary gastric cancer patients, who were treated at the National Cancer Center Hospital in Tokyo between 1968 and 1989. The software can calculate the probability of lymph node involvement in stations No. 1–16., based on various prognostic factors. MCP was first validated in Japanese patients and the program was able to predict LN involvement in 94% (47). The accuracy was increased from 66% to 93% by using an artificial neural network (48).

Our previous study successfully demonstrated a similarly high level of reliability of MCP, reaching 90.2% of sensitivity, 63.3% of specificity, and 78.4% of accuracy (49). The prediction of LN metastases was shown to be superior to the standard pre-operative imaging techniques.

Traditionally the MCP was a great tool to determine the expected long-term oncological outcomes. Its usefulness was demonstrated by Hundahl (39) after the Intergroup 0116 Trial. He defined the term Maruyama Index (MI) first to measure the unresected regional nodal disease. Later, Hundahl made a blinded reanalysis of the Dutch D1-D2 trial by the autopsy ﬁndings. He demonstrated, that MI < 5 or a low MI surgery is associated with enhanced regional control and survival (50). Based on previous data, the Maruyama Index of less than 5 had a better impact on survival than any D-level guided surgery.

Dikken et al. proved the prognostic significance of low MI in a 2-year survival rate (82% vs. 59%) (51), as did Sachdev, who represented the correlation between lower MI values and higher survival rates, as continuous (*P* < 0.02) and categorical (*P* < 0.04) variables (52).

In light of contemporary oncological treatment, these results are worth reassessing. By predicting the probability of lymph node involvement better than any conventional imaging modalities, it still has the potential to indicate the necessity for neoadjuvant oncological treatment and also helps the surgeon to focus on key lymph node stations during the subsequent lymphadenectomy.

## Discussion

Gastric cancer is still a major cause of cancer-related deaths. Despite the advances in prevention, diagnostics, and therapy, it accounts for 768 793 deaths worldwide. A crucial challenge is to translate recent discoveries in molecular biology into oncological treatment for patients with gastric cancer.

Surgery is still the most important modality to properly stage and eradicate gastric cancer. For most patients, performed with curative intention, is the best chance for long-term survival. The type and extent of the operation are greatly influenced by the histological type, location, and stage of the tumor.

The concept of hybrid laparo-endoscopic techniques, sentinel node navigation surgery, and utilization of the Maruyama Computer Program are significant components of stage-adapted gastric cancer surgery. Centralization and application of national guidelines could improve both the surgical and the oncological outcomes.

The widespread use of neoadjuvant therapy and its effect on the lymphatic drainage of tumors is mostly unknown, as are the future benefits of information regarding the extent of lymph node dissection.
